# Cough

**Published:** 1876-04

**Authors:** 


Cough.—For the irritating cough that is so harassing to many patients, nothing is better than frequent small draughts of warm water; and when the throat is inflamed, or the tonsils swollen so that deglutition is painful, a wet napkin covered with a dry cloth should be put around the neck and worn until relieved, rewetting the cloth as often as it becomes dry. It should be wet in cool water.—*Med. Brief.*

